# Supplementary material for: Radiologists’ Perspectives on AI Integration in Mammographic Breast Cancer Screening: A Mixed Methods Study
Source: Cancers (Basel). 2025 Oct 30;17(21):3491. doi: 10.3390/cancers17213491 (PMC12607412; doi:10.3390/cancers17213491)
Supplement: Supplementary file 1 [file cancers-17-03491-s001.zip › cancers-3939073-supplementary.pdf]

Supplementary Material S1: Quantitative survey questions

AI Diagnostic Capabilities

1. How would you rate AI's diagnostic capabilities compared to human radiologists?

    - o Comparable
    - o Worse
    - o Better

2. In your opinion, can AI replace one human reader in mammography screening?

    - o Yes
    - o No
    - o Maybe

3. In your opinion, will AI be useful as a companion reader/reader aid in mammography screening?

    - o Yes
    - o No
    - o Maybe

4. In your opinion, will AI be useful for triaging in mammography screening?

    - o Yes
    - o No
    - o Maybe

Confidence Levels

5. On a scale of 1 to 10, how confident are you in using AI as a companion reader?
    a. (1-10 scale)
6. On a scale of 1 to 10, how confident are you in making correct diagnoses with AI assistance?
    a. (1-10 scale)

Access to AI

7. Who do you believe should have access to AI in the screening program? (Select all that apply)
    a. First Reader

b. Second Reader

c. Referee/Arbitration Panel

Conflict Resolution

8. In cases of conflicting opinions between radiologists and AI, whose opinion should prevail?

    a. Radiologist’s Opinion

    b. AI’s Opinion

    c. Discussion Among Radiologists

Utility of AI Features

9. How would you rank the importance of the following features of diagnostic AI? (Rank from 1 to 6, with 1 being most important)

    a. Heat map or bounding boxes on the region of suspicion

    b. Ability to provide a discrete opinion

    c. Mammographic risk scoring

    d. Triaging of mammograms

    e. Breast density estimation

    f. Radiological report generation

Evidence Confidence Levels

10. What evidence would you need to use AI? (1 = Least required, 10 = Most required)

    a. Testing the product with local data

    b. National guidelines

    c. Studies using a nationally representative data set

    d. Independent prospective studies

    e. Independent retrospective single-site studies

    f. Vendor's internal analysis of the product

<u>Supplementary Material S2: Semi-structured interview guide</u>

Introduction:

- Explain the purpose of the interview and ensure confidentiality.
- Build rapport with participants:
    - o Disclaimer: All opinions shared in this interview should be pertaining to the use of AI in mammography to facilitate the breast cancer screening process.
    - o Demographics:
        - ▪ Age.
        - ▪ Gender.
        - ▪ No of years working as a radiologist.
        - ▪ Duration of exposure to AI.
    - o Could you share with me your first impression of AI?
    - o Share experience on use of **any** AI software for mammographic detection of breast cancer?

Perceived Usefulness (Performance, effort expectancy):

- What are your thoughts on the implementation of AI in the current breast cancer screening workflow?
- Do you think AI can replace humans in the reading of mammograms?
- How do you think the implementation of AI in a clinical setting will affect your clinical workflow?
- Which AI assistant feature do you find the most useful and why?
- How do you feel about AI being used in different capacities, such as:
- Do you think the diagnostic ability of AI in detecting cancers in mammograms is comparable to a human radiologist? Why or why not?

Perceived Ease of Use (Social influence, facilitating conditions):

Some participants have voiced concerns with regards to integrating the use of AI into their current work flow,

- How easy or difficult do you find integrating AI tools into your current workflow?
- What challenges have you faced in using AI tools?
- Do you have any suggestions to facilitate this implementation process?

Behavioural Intention to Use:

- How likely are you to adopt AI tools in your practice in the future?
- What factors influence your decision to use or not use AI tools?

User behaviour:

- Have you used AI tools in your practice? If so, how frequently?
- What has been your experience with these tools?

Additional Insights:

- In the hypothetical situation, if there is a differing opinion between your medical judgment and the AI judgment, which should be followed?

Closing Questions:

- If you could envision a future with AI integrated into the workflow for breast cancer screening, what would it look like?
- Is there anything else you would like to add about AI in breast cancer screening?

Supplementary Material S3: Good reporting of a mixed-methods study (GRAMMS) checklist

Checklist Item	Description	Page Information
1. **Justification for using a mixed methods approach**	Explain why a mixed methods approach was necessary to address the research question.	2
2. **Design: Purpose, Priority, and Sequence of Methods**	- **Purpose**: State the goal of combining qualitative and quantitative data.<br>- **Priority**: Indicate whether one method had precedence or both were equally weighted.<br>- **Sequence**: Describe the order of applying the methods (e.g., sequential or concurrent).	2-3
3. **Methods: Sampling, Data Collection, and Analysis**	- **Sampling**: Specify the sampling strategies used (e.g., random sampling, purposive sampling).<br>- **Data Collection**: Outline how data were gathered (e.g., surveys, interviews).<br>- **Analysis**: Describe the analysis techniques for each method (e.g., statistical analysis for quantitative, thematic analysis for qualitative).	2-4
4. **Integration: Where, How, and Who**	- **Where**: Indicate where integration occurred (e.g., in analysis or interpretation).<br>- **How**: Describe the process of integration (e.g., merging, embedding, or comparing results).<br>- **Who**: Identify participants involved in integration (e.g., researchers, analysts).	4-5
5. **Limitations of One Method due to the Other**	Mention any constraints or challenges faced by one method as a result of using the other (e.g., timing, resource constraints).	6
6. **Insights Gained from Mixing Methods**	Describe any unique insights or findings that emerged from combining qualitative and quantitative data.	5
